# Protection of p53 wild type cells from taxol by nutlin-3 in the combined lung cancer treatment

**DOI:** 10.1186/1471-2407-10-57

**Published:** 2010-02-23

**Authors:** Sergey V Tokalov, Nasreddin D Abolmaali

**Affiliations:** 1 OncoRay - Center for Radiation Research in Oncology, Medical Faculty Carl Gustav Carus, Dresden University of Technology, Fetscherstraße 74/P.O. Box 86, 01307 Dresden, Germany

## Abstract

**Background:**

Mutations within the tumor suppressor *TP53 *gene are one of the most common genetic alterations present at high frequency in human tumors and have been shown to be associated with resistance to radio-chemotherapy. The lack of the wild type *TP53 *gene in cancer cells could be exploited for therapeutic advantage using a sequence of two antagonistic drugs. The aim of this study was to selectively kill p53 deficient cells (FaDu and H1299) by taxol and to protect p53 wild type cells (A549) by the prior administration of nutlin-3 in comparison to certain known anticancer drugs (5-fluorouracil, camptothecin, roscovitine).

**Methods:**

Cytotoxic and cytostatic properties of 5-fluorouracil, camptothecin, roscovitine and nutlin-3 administrating alone or in combination with taxol were investigated in vitro by flow cytometry.

**Results:**

It was found that nutlin-3 induced growth arrest and protected A549 cells from taxol. FaDu and H1299 cells responded to the same treatments with mitotic arrest and massive apoptosis. Other compounds (5-fluorouracil, camptothecin and roscovitine) revealed weaker selectivity and elevated toxicity in comparison to nutlin-3.

**Conclusions:**

We propose a therapeutic strategy protecting normal cells from taxol while increasing apoptosis selectively in p53-deficient cells using nutlin-3.

## Background

Cancer is a complex family of diseases, characterized by the deregulation of normal control pathways for cellular growth. Lung cancer (LC) is the leading cause of death among human malignancies and is among the most threatening of them due to its disappointing response to therapy [1]. Development of LC, which can be separated roughly into small cell lung cancer (SCLC) or non-small cell lung cancer (NSCLC), involves multiple genetic abnormalities. One of the most common changes on this way is mutation in the tumor suppressor *TP53 *gene with a mutations frequency of 50% and 70% in NSCLC and SCLC, respectively [2-4]. Such genetic abnormality is shown to be associated with a poorer survival prognosis and increased cellular resistance to therapy [5-7]. Thus, there is an urgent need for development of target-driven novel class of anti-cancer drug against this deadly disease.

The discovery of new cancer-related therapeutic targets is mainly based on the identification of genes involved in pathways selectively exploited in cancer cells [8,9]. For example, the lack of wt p53 (the product of *TP53 *gene) in cancer cells can be utilized for therapeutic advantage by selective killing of p53 deficient (p53^-/-^) cancer cells and by protecting p53 wild type cells (p53^wt^) at normal proliferation rates using antagonistic drugs [10,11]. It was demonstrated that certain anticancer drugs could selectively arrest p53^wt ^cells in G_1 _or G_2 _phases of the cell cycle by activation of the p53 pathway and thereby protects them from antimitotic agent. E.g. taxol, which simultaneously kills and/or blocks p53^-/- ^cancer cells during mitosis [12,13].

However, genotoxic drugs can trigger multiple molecular events including activation of p53-independent checkpoints and thus may partially protect the cancer cells during chemotherapy [3]. This can be avoided by using agents targeted specifically at the p53 pathway. In proliferating cells that are not subjected to stress, p53 level is tightly controlled by its negative regulator MDM2, which binds p53 and modulates its transcriptional activity and stability [3,14-16]. MDM2 is an E3 ubiquitin ligase that binds the tumor suppressor and facilitates its ubiquitin-dependent degradation [17]. The MDM2 binding domain overlaps with the transcriptional activation domain of p53, and therefore MDM2 binding also inhibits the transcriptional activity of p53, thus effectively impairing its function [18]. Disruption of the p53-MDM2 interaction, therefore, provides an attractive strategy for activating p53. It was shown that nutlin-3 could selectively disrupt the interaction between p53 and MDM2 [19] inducing cell cycle arrest in normal murine and human cells [3,14-16,20] cells without initiation of apoptosis. This presents unique opportunities for p53-dependent modulation of the cell cycle of the proliferating p53^wt ^cells of the intact surrounding tissues to protect them from the taxol during chemotherapy of p53^-/- ^tumors [21].

In this context, the goal of our work was to evaluate the effectiveness of the MDM2 antagonist nutlin-3 in comparison to certain anticancer drugs (5-fluorouracil, camptothecin, roscovitine) with known cytostatic effects to protect proliferating p53^wt ^cells from taxol in the combined cell cycle associated therapy leading to selective killing of p53^-/- ^cells.

## Methods

### Cell culture

Established human cell lines of different tumor entities, i.e. near triploid (~3C) NSCLC cell line A549 presenting wild type *TP53 *gene, near hexaploid (~6C) NSCLC cell line H1299 with *TP53*-null gene and near triploid (~3C) pharyngeal squamous-cell carcinoma (PSCC) cell line FaDu presenting mutated *TP53 *gene from DSMZ (Germany) were used in this study. Cells were cultured in Dulbecco Modified Eagle Medium routinely supplementing with 10% heat-inactivated fetal calf serum (Gibco, France) and incubated at 37°C in humidified 5% CO_2 _atmosphere with media replacement every 2 days. When the cell cultures reached 90% confluence they were harvested using 0.25% trypsin and 1 mM EDTA solution (Sigma, Germany) and reseeded in new flasks at a density of 10-15 × 10^3^cells/cm^2^. Cell counts in the samples were determined with a coulter counter (CASY, Model TTC, Schärfe System, Germany).

### Flow cytometry

To have a possibility for comparison of our results with previous findings [3,12,14-16,19,20,22-28] and taking into account that in solid tumor-derived cell lines p53-dependent apoptosis is usually delayed for 24 h [19] the cells were placed in culture medium (2 × 10^5^cells/mL) 1 day before exposure to 5-fluorouracil (1, 3, 10 and 30 μM), camptothecin (10, 30, 100 and 300 nM), nutlin-3 (1, 3, 10 and 30 μM), roscovitine (1, 3, 10 and 30 μM) and taxol (1, 3, 10, 30 and 100 nM). All compounds were pursued from Sigma (Germany). To test an advantage of the nutlin-3 in the selective protect p53^wt ^A549 cells from taxol, all cell cultures according to [3,29] were incubated for a 24 hours period in parallel with 5-fluorouracil (3 μM), camptothecin (10 nM), roscovitine (10 μM) and nutlin-3 (3 μM) respectively. Then, taxol (10 nM) was added and cell cultures were incubated for another 24 hours period. Because the stock solutions (×1000) of the test compounds were prepared in dimethylsulfoxid (DMSO, Sigma, Germany), all cultures including the control samples were made 0.1% in DMSO. After 24 hours of appropriate treatment cells were removed from the culture and prepared for analysis by flow cytometry as previously described [30]. Cells were briefly washed with phosphate-buffered saline (PBS) and centrifuged at 100 g for 10 min. The cell pellet was re-suspended in 100 μl of PBS, fixed in 70% (vol/vol) ethanol by adding 1 ml of cold (-20°C) ethanol and stored overnight at -20°C. The cells were spun down again and the pellet was suspended in 1.5 ml of PBS at room temperature. After centrifugation the cell pellet was suspended in 1 ml DNA staining solution containing 50 μg PI and 0.2 mg RNase (both Sigma, Germany) and incubated for at least 45 min at room temperature in the dark. About 2 × 10^5 ^cells per sample were analyzed by flow cytometry (CyFlow, Partec, Germany). The excitation wavelength was 488 nm, and red fluorescence (>590 nm for PI) was recorded. In addition, the parameters for forward scatter (FSC) and side scatter (SSC) were determined. For each variable (exposure condition, culture period etc.) a minimum of 6 samples were quantified. The flow cytometer was calibrated with 2.5 μm polyfluorescent beads (AlignFlow, Molecular Probes, Eugene, OR) before each series of measurements.

The fractions of apoptotic cells with sub-G_1 _DNA content as well as the fractions of cells in G_0 + 1_, S and G_2 _+ M phases of the cell cycle were quantified according to the level of measured fluorescence, FSC and SSC using a CyFlow software (Partec, Germany).

### Statistics

The experimental results are expressed as the mean ± standard deviation (mean ± s.d.) of 6 independent experiments. Analysis of variance (ANOVA) was performed.

## Results

In vitro cultures of exponentially growing A549, H1299 and FaDu intact cell cultures had different cell cycle distributions with 62 ± 1%, 46 ± 1% and 59 ± 1% in G_0 + 1_, 26 ± 1%, 36 ± 1% and 26 ± 1% in S and 12 ± 1%, 18 ± 1% and 15 ± 1% in G_2 _+ M phases respectively. In the first part of investigation all cell cultures were incubated for a 24 hours period in parallel with dissimilar concentrations of 5-fluorouracil, camptothecin, nutlin-3, roscovitine and taxol with respect to the toxicity and cell cycle effects of test compounds.

No perturbations of the cell cycle were registered in the control cell cultures. With increasing concentrations of the tested compounds the cell cycle progression was affected in varying degrees (Table 1).

**Table 1:** Effects of test substances on proliferation of certain cancer cells.

**The distribution of cells in different cell cycle phases (%)^1^**												
	**A549**				**FaDu**				**H1299**			
	**<2C**^2^	**G_0 + 1_**	**S**	**G_2 _+ M**	**<2C**	**G_0 + 1_**	**S**	**G_2 _+ M**	**<2C**	**G_0 + 1_**	**S**	**G_2 _+ M**
5-fluorouracil (μM)												
1	2 ± 1	62 ± 2	27 ± 1	11 ± 1	2 ± 1	51 ± 3	31 ± 2	16 ± 1	2 ± 1	56 ± 2	31 ± 2	15 ± 1
3	2 ± 1	57 ± 2	**33 ± 1**	10 ± 1	2 ± 1	**55 ± 3**	**26 ± 2**	19 ± 1	2 ± 1	**52 ± 2**	**34 ± 2**	14 ± 1
10	2 ± 1	**31 ± 2**	**61 ± 2**	8 ± 2	2 ± 1	**28 ± 1**	**52 ± 1**	20 ± 1	2 ± 1	**52 ± 2**	**32 ± 2**	16 ± 2
30	3 ± 1	**46 ± 2**	**47 ± 2**	7 ± 2	3 ± 1	48 ± 2	**44 ± 1**	18 ± 1	5 ± 1	**50 ± 2**	**33 ± 2**	17 ± 2
Camptothecin (nM)												
10	2 ± 1	**42 ± 4**	24 ± 2	**28 ± 3**	6 ± 2	**18 ± 1**	**24 ± 2**	**58 ± 3**	2 ± 1	**17 ± 4**	**40 ± 9**	**43 ± 9**
30	**8 ± 2**	**16 ± 2**	**68 ± 4**	16 ± 4	**10 ± 3**	**6 ± 1**	**77 ± 3**	17 ± 3	**12 ± 2**	**11 ± 3**	**69 ± 3**	19 ± 2
100	**32 ± 4**	**14 ± 2**	**69 ± 4**	17 ± 2	**17 ± 2**	**21 ± 3**	**68 ± 4**	**11 ± 1**	**17 ± 3**	**22 ± 1**	**56 ± 4**	**22 ± 3**
300	**45 ± 6**	**45 ± 4**	**45 ± 4**	10 ± 2	**15 ± 3**	**40 ± 3**	**50 ± 3**	**10 ± 2**	**15 ± 2**	**35 ± 3**	**40 ± 4**	**25 ± 2**
Nutlin-3 (μM)												
1	2 ± 1	61 ± 2	28 ± 2	11 ± 1	2 ± 1	40 ± 4	38 ± 3	22 ± 3	2 ± 2	57 ± 1	29 ± 2	14 ± 1
3	2 ± 1	**71 ± 2**	**13 ± 1**	**16 ± 1**	2 ± 1	44 ± 2	34 ± 2	22 ± 2	2 ± 1	57 ± 3	25 ± 2	18 ± 2
10	2 ± 1	**69 ± 1**	**7 ± 3**	**24 ± 3**	4 ± 1	45 ± 3	34 ± 2	21 ± 2	4 ± 1	54 ± 4	27 ± 4	19 ± 2
30	**5 ± 1**	**68 ± 2**	**6 ± 1**	**26 ± 2**	**8 ± 1**	**48 ± 3**	**32 ± 2**	20 ± 2	**7 ± 1**	63 ± 2	**19 ± 2**	18 ± 1
Roscovitine (μM)												
1	2 ± 1	60 ± 2	24 ± 2	16 ± 1	2 ± 1	41 ± 4	39 ± 4	20 ± 2	2 ± 1	**47 ± 1**	**36 ± 3**	17 ± 2
3	2 ± 1	59 ± 4	20 ± 3	**21 ± 1**	2 ± 1	43 ± 1	32 ± 1	**25 ± 1**	2 ± 1	**52 ± 1**	**33 ± 1**	15 ± 2
10	5 ± 1	59 ± 4	20 ± 3	**21 ± 1**	4 ± 1	**38 ± 2**	**27 ± 2**	**35 ± 2**	4 ± 2	**49 ± 2**	29 ± 1	**22 ± 1**
30	**10 ± 4**	**51 ± 5**	24 ± 3	**24 ± 3**	**10 ± 3**	**34 ± 3**	30 ± 2	**36 ± 2**	**11 ± 4**	**43 ± 3**	26 ± 2	**31 ± 3**
Taxol (nM)												
1	**9 ± 2**	60 ± 4	25 ± 4	15 ± 2	21 ± 2	39 ± 3	34 ± 6	27 ± 4	**13 ± 6**	48 ± 6	30 ± 3	22 ± 5
3	**36 ± 5**	**41 ± 3**	32 ± 5	**27 ± 2**	**30 ± 5**	**22 ± 4**	35 ± 5	**43 ± 7**	**35 ± 6**	**40 ± 1**	**40 ± 1**	**20 ± 1**
10	**35 ± 6**	**22 ± 4**	29 ± 3	**51 ± 6**	**33 ± 6**	**12 ± 2**	**19 ± 4**	**69 ± 4**	**42 ± 5**	**34 ± 4**	**34 ± 4**	**32 ± 7**
30	**16 ± 4**	**15 ± 2**	**17 ± 3**	**68 ± 5**	**16 ± 3**	**4 ± 1**	**11 ± 2**	**85 ± 2**	**44 ± 4**	**18 ± 1**	**36 ± 4**	**46 ± 7**
100	**10 ± 2**	**18 ± 4**	**18 ± 3**	**64 ± 5**	**8 ± 2**	**5 ± 1**	**9 ± 3**	**87 ± 3**	**30 ± 4**	**21 ± 3**	23 ± 3	**55 ± 2**
Control												
- ^3^	2 ± 1	62 ± 1	26 ± 1	12 ± 1	2 ± 1	46 ± 2	36 ± 1	18 ± 1	2 ± 1	59 ± 2	26 ± 2	15 ± 1

The data show the effects of the test substances on the cell cycle of A549, FaDu and H1299 cells after exposure for 1 day (average ± SD of 6 independent experiments). Significant differences (p < 0.05) to control cultures are indicated in bold print.

^1^The distribution of cells in different cell cycle phases was calculated from the population of cycling cells (G_0 + 1 _+ S + G_2 _+ M = 100%).

^2^The percentage of hypodiploid cells (<2C DNA content) was calculated from the total number of cells.

^3^Since the test compounds were dissolved in a DMSO stock solution, control cells cultures were made in 14 mM (0.1%) DMSO.

5-fluorouracil provoked a dose dependent delay in S phase (3-30 μM, p < 0.05) in all three cell lines independent of their p53 status. It was accompanied by decreasing percentage of G_0 + 1 _cells in A549 (10-30 μM, p < 0.05), FaDu (10 μM, p < 0.05) and H1299 (3-30 μM, p < 0.05).

Camptothecin was the most toxic of the test compounds used showing a dose dependent increase of apoptotic cell proportion (p < 0.05), causing cell cycle arrest at S phase (p < 0.05) and reduction of G_0 + 1 _cells proportion (p < 0.05) at concentrations of 30-300 nM in all three cell lines independent of their p53 status.

Nutlin-3 at concentrations of 3-30 M caused reduction of S cells proportion (p < 0.05) with increase of percentage in G_0 + 1 _(p < 0.05) and G_2 _+ M phases (p < 0.05) in p53^wt ^A549 cells. No remarkable changes in the cell cycle distribution were registered in p53^-/- ^FaDu and H1299 cells. These observations were accompanied by an increasing proportion of apoptotic cells (30 μM, p < 0.05) in all cell lines used.

Roscovitine caused increase of A549 (3-30 μM, p < 0.05), FaDu (3-30 μM, p < 0.05) and H1299 (10-30 μM, p < 0.05) cells proportion at G_2 _+ M phase. Decreases of G_0 + 1 _cells percentage were registered in A549 (30 μM, p < 0.05), FaDu (10-30 μM, p < 0.05) and H1299 (1-30 μM, p < 0.05) cells. These changes were accompanied by the increasing proportion of apoptotic cells (30 μM, p < 0.05) in all three cell lines.

Taxol caused dose dependent accumulation of cells arresting at the G_2 _+ M phase accompanied by the reduction of the proportion of the cells in G_0 + 1 _and S phases (3-100 μM, p < 0.05) independent of their p53 status. At lower concentrations (1-30 nM, p < 0.05), a large fraction of cells was released from the block in 24 hours and became apoptotic. At higher concentrations (30-100 nM) the proportion of apoptotic cell was reduced due to elongation of G_2 _+ M block.

**Figure 1:**
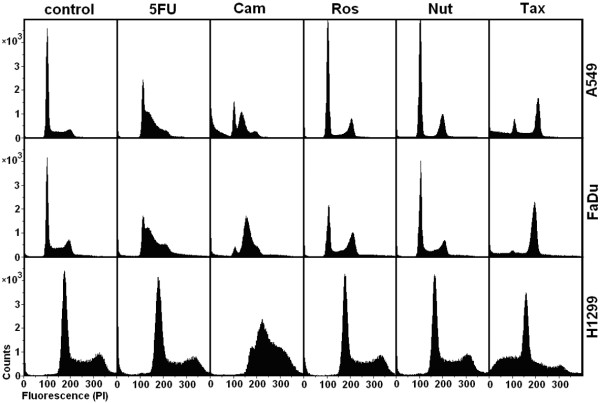
**The effect of certain compounds on cell cycle distribution**. Cell cycle distribution in 24 hours after administration of DMSO (0.1%, control), 5-fluorouracil (5FU, 3 μM), camptothecin (Cam, 10 nM), roscovitine (Ros, 10 μM), nutlin-3 (Nut, 3 μM) and taxol (Tax, 10 nM).

**Figure 2:**
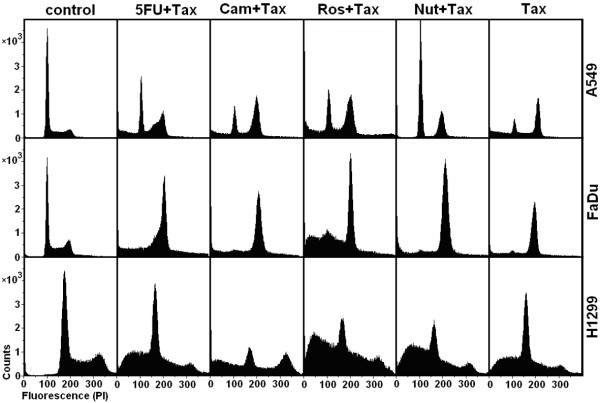
**Cell cycle distribution in 24 hours after combined treatment**. Cell cycle distribution in 24 hours after administration of DMSO (0.1%, Control), 5-fluorouracil (5FU, 3 μM), camptothecin (Cam, 10 nM), roscovitine (Ros, 10 μM), nutlin-3 (Nut, 3 μM), followed by administration of taxol (Tax, 10 nM) for another 24 hours.

To test the ability of the analyzed compounds to selectively protect p53^wt ^A549 cells and to selectively kill p53^-/- ^H1299 and FaDu cells by taxol, all cell cultures were incubated for a 24 hours period in parallel with DMSO (0.1%, control), 5-fluorouracil (3 μM), camptothecin (10 nM), roscovitine (10 μM), nutlin-3 (3 μM) and taxol (Tax, 10 nM) respectively (Figure 1). Then, taxol (10 nM) was added and cell cultures were incubated for another 24 hours period. It was found (Figure 2) that the administration of taxol alone increased the proportion of apoptotic cells in A549 (35 ± 6%, p < 0.001), FaDu (33 ± 4%, p < 0.001) and H1299 (42 ± 5%, p < 0.001) cell lines. Pretreatment with nutlin-3 protected p53^wt ^A549 cells (but not p53^-/- ^FaDu and H1299 cells) from taxol, dramatically reduced the proportion of apoptotic cells (2 ± 1%, p < 0.001; 29 ± 2%, p < 0.001; 52 ± 4%, p > 0.05, correspondently) and revealed the similar distribution through the cell cycle, as it was registered after the administration of nutlin-3 alone.

Pretreatment with roscovitine also protected some of A549. However, roscovitine increased proportion of apoptotic A549 cells as well as protected some of p53^-/- ^FaDu cells due to a block in G_2 _+ M phase. As a result proportion of apoptotic A549, FaDu and H1299 cells reduced to 20 ± 2%, p < 0.001; 21 ± 2%, p < 0.001; 49 ± 3%, p > 0.05, correspondently. The other compounds analyzed (5-fluorouracil, camptothecin) revealed low selectivity and demonstrated high toxicity as well.

## Discussion

Our findings in cell cycle specific interaction of anticancer drugs (5-fluorouracil, camptothecin, nutlin-3, roscovitine, taxol) generally validate previous reports in the literature. So, it is confirmed that treatment of cells with 5-fluorouracil widely using in the treatment of a range of cancers leads to an accumulation of cells in S-phase and induces p53 apoptosis [22]. Naturally occurring cytotoxic alkaloid camptothecin reveals the highest toxicity from the test compounds due to the possibility irreversibly binding to the DNA-topoisomerase I complex independent of 53 status of cells [23]. Roscovitine inhibiting cyclin-dependent kinases via direct competition in the ATP-binding site [26] provokes G_2_-M arrest and at the highest using doses induces apoptosis in all of used cells. Following binding to B-tubulin microtubule-targeted agent taxol causes mitotic arrest and apoptosis in variety of cancer cells [27]. Such blocked cells could be arrested during next several days of culture. In contrast, at lower concentrations a large fraction of cells could release from the block in 24 hours and became apoptotic [28]. Our study in agreement with previous findings [31] showed that nutlin-3 in low concentrations (1-10 μM) induced depletion of the S-phase fraction, causing arrest at G_1_/S and/or G_2_/M phases in p53^wt ^A549 cells without apparent apoptosis. Last may be explained by the known fact that the induction of apoptotic genes alone sometimes is not sufficient to provoke apoptosis, as the high levels of cell cycle inhibitor, such as p21 dominantly lead to the cell cycle arrest [24]. This was supported using MI-43 (another compound that disrupted Mdm2-p53 binding) which at low concentrations induced remarkable induction of p53 leading to 90% cells arrested of A549 cells at the G_1 _phase without apparent apoptosis, although Puma and Noxa were also induced. However, at higher drug concentration cells underwent apoptosis even with a moderate further increase of Puma and Noxa[25].

Previous studies show that elevated concentrations of nutlin-3 induced p53- and p21-dependent cell cycle arrest and p53-dependent cell death in different p53^wt ^tumor cell lines including A549 [15,16,18,19,21,25,27,29,31]. The p53 activation by nutlin-3 has been shown to lead cell cycle arrests in normal human [3,15] fibroblasts, endothelial [16] and epithelial [20] cells without initiation of apoptosis. Therefore, NSCLC cell line A549 seems to be more sensitive to the induction of apoptosis through the activation of p53 pathway and our results might be translated to the control cell lines mentioned above.

Taxol and other mitotic chemotherapeutics are frequently used together with genotoxic drugs activating the p53 pathway in wild-type p53 cells via genotoxic stress [10]. However, the usefulness of DNA damaging agent is limited by their ability to activate p53-independent checkpoint mechanism in cancer cells with mutant p53 [11]. The nutlin-3 works solely through stabilization and activation of p53 gene. Therefore, protection by nutlin-3 is strictly dependent on the p53 status of the cells [21]. Using MDM2 antagonist nutlin-3 as selective activator of p53 pathway, we have shown that induction of cell cycle arrest can protect p53^wt ^NSCLC (A549) cells from the cytotoxicity of taxol selectively killing of p53^-/- ^PSCC cells (FaDu) and p53^-/- ^NSCLC (H1299) cells. Our results confirmed previously reported synergistic interaction of the nutlin-3/taxol treatment of the p53^-/- ^cancer cells and protection of normal proliferating fibroblasts or p53^wt ^colon cancer cells [3].

Although the experiments described in this report use taxol as a mitotic inhibitor, nutlin-3 halted cell cycle progression at the G_1_/S and G_2_/M phases and can thus attenuate the activity of S-phase- and M-phase-specific drugs. For example, treatment of normal proliferating fibroblasts or keratinocytes with nutlin-3 protects these cells against gemcitabine and Ara-C killing proliferating p53-/- cancer cells in S-phase [29].

From other side, previous findings have suggested that nutlin-3 at higher concentrations might also offer a new therapeutic option to patients with tumors that express wild-type p53 either as single agent (Vassilev, 2005; Tovar et al., 2005) or as combination therapy (Kojima et al., 2005; Stuhmer et al., 2005). Nutlins have been shown to enter multiple types of cultured cells and inhibit the p53-MDM2 interaction in the cellular context with a high degree of specificity, resulting in the p53 stabilization, p21 induction, cell cycle arrest in G1 and G2 phases, apoptosis and growth inhibition of proliferating cancer cells [19]. It is interesting that treatment of nude mice with nutlin-3 has been shown might effectively inhibit the growth of tumor xenografts without revealing of overt toxicity, thus suggesting that normal tissues may have higher tolerance to p53 activation [21].

Our findings open possibility to establish a genetically determined strategy in the lung cancer treatment. Depending of genetic status of tumor nutlin-3 might be administrated as an agent for activation of the p53 pathway resulting in p53 stabilization, p21 induction, cell cycle arrest in G_1 _and G_2 _phases, apoptosis and growth inhibition of cancer cells or in combination with taxol as a compound protecting surrounding normal tissue. However, an inter-organ metabolism of nutlin-3 might be critical to its absorption, distribution and modification of the resulting therapeutic effects in vivo.

Therefore, additional in vivo experiments in which the therapeutic role of nutlin-3 is assessed on multiple normal tissues in human tumor xenografts with different p53 status may help to better understand the clinical potential of this approach.

## Conclusions

The therapeutic strategy protecting normal cells from taxol, while selectively increasing apoptosis in p53-deficient cells is proposed. Nutlin-3 application opens possibility to establish a genetically determined strategy in the lung cancer treatment. However, metabolism of used compounds in different tissues should be taken into account for evaluation of therapeutic effects of this approach.

## Competing interests

The authors declare that they have no competing interests.

## Authors' contributions

SVT carried out the literature search, designed the study performed *in vitro *cell culture, cell treatment, flow cytometry, statistical analysis and prepared the manuscript. NDA coordinated the study and drafted the manuscript.

## Pre-publication history

The pre-publication history for this paper can be accessed here:

http://www.biomedcentral.com/1471-2407/10/57/prepub
